# Similar insulin regulation of splanchnic FFA and VLDL-TG in men with nonalcoholic hepatic steatosis and steatohepatitis

**DOI:** 10.1016/j.jlr.2024.100580

**Published:** 2024-06-18

**Authors:** Jeyanthini Risikesan, Sara Heebøll, Indumathi Kumarathas, Esben Søndergaard, Rakel F. Johansen, Steffen Ringgaard, Niels K. Aagaard, Thomas D. Sandahl, Gerda E. Villadsen, Lars C. Gormsen, Jan Frystyk, Michael D. Jensen, Henning Grønbæk, Søren Nielsen

**Affiliations:** 1 Steno Diabetes Center Aarhus, Aarhus University Hospital (AUH), Aarhus, Denmark; 2 Department of Endocrinology and Internal Medicine, AUH, Aarhus, Denmark; 3 MR Research Centre, Aarhus University, Aarhus, Denmark; 4 Department of Hepatology and Gastroenterology, AUH, Aarhus, Denmark; 5 Department of Clinical Medicine, Aarhus University, Aarhus, Denmark; 6 Department of Nuclear Medicine and PET Centre, AUH, Aarhus, Denmark; 7 Department of Endocrinology, Odense University Hospital, Odense, Denmark; 8 Endocrine Research Unit, Mayo Clinic, Rochester, MN, USA

**Keywords:** nonalcoholic fatty liver disease, liver, free fatty acids, VLDL, triglycerides, visceral adipose tissue, lipolysis and fatty acid metabolism, lipoproteins/kinetics, insulin

## Abstract

This study aimed to determine whether obese men with nonalcoholic fatty liver disease (NAFLD) display differences between those with simple steatosis versus steatohepatitis (NASH) in splanchnic and hepatic FFA and VLDL-triglycerides (VLDL-TG) balances. The study involved 17 obese men with biopsy-proven NAFLD (9 with NASH and 8 with simple steatosis). We used hepatic vein catheterization in combination with [^3^H]palmitate and [^14^C]VLDL-TG tracers to measure splanchnic palmitate and VLDL-TG uptake and release rates during basal and hyperinsulinemic conditions. Indocyanine green was used to measure splanchnic plasma flow. Splanchnic palmitate uptake was similar in the two groups and significantly reduced during hyperinsulinemia (NASH: 62 (48–77) versus 38 (18–58) μmol/min; simple steatosis: 62 (46–78) versus 45 (25–65) μmol/min, mean (95% CI), basal versus clamp periods, respectively, *P* = 0.02 time-effect). Splanchnic palmitate release was also comparable between groups and nonsignificantly diminished during hyperinsulinemia. The percent palmitate delivered to the liver originating from visceral adipose tissue lipolysis was similar and unchanged by hyperinsulinemia. Splanchnic uptake and release of VLDL-TG were similar between groups. Hyperinsulinemia suppressed VLDL-TG release (*P* <0.05 time-effect) in both groups. Insulin-mediated glucose disposal was similar in the two groups (*P* = 0.54). Obese men with NASH and simple steatosis have similar splanchnic uptake and release of FFA and VLDL-TG and a similar proportion of FFA from visceral adipose tissue lipolysis delivered to the liver. These results demonstrate that the splanchnic balances of FFA and VLDL-TG do not differ between obese men with NASH and those with simple steatosis.

## Graphical Abstract

**Figure:**
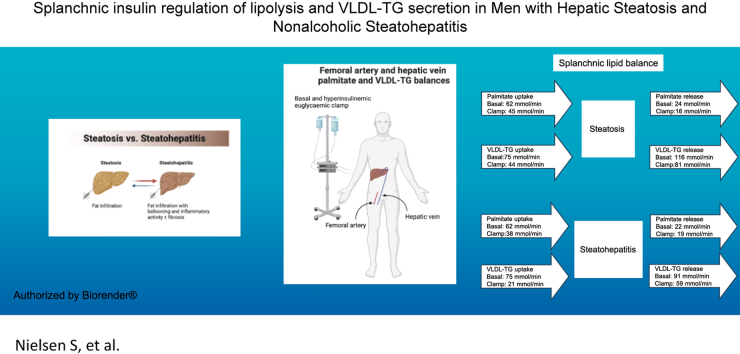

Nonalcoholic fatty liver disease (NAFLD) is associated with obesity with preferential visceral adipose tissue (VAT) accumulation and dyslipidemia. A subgroup of patients of up to 25% in follow-up studies are believed to progress from simple steatosis to nonalcoholic steatohepatitis (NASH) (1, 2) with hepatocyte ballooning and lobular inflammation with or without fibrosis (3) which predicts a greater risk for progressive liver disease, type 2 diabetes, and cardiovascular disease. (4, 5, 6, 7) The increased intrahepatic triglyceride (IHTG) content ultimately depends on an imbalance between hepatic lipid storage (FFA and lipoproteins/remnants uptake and de novo lipogenesis) and utilization (hepatic fatty acid oxidation, ketone body production, and export as very low-density triglyceride lipoproteins (VLDL-TG)) and it is conceivable that an inability of the liver to balance lipid influx and outflux differ between subjects with simple steatosis and NASH.

Our previous findings show that obese individuals have greater splanchnic uptake of FFA and that the proportion of hepatic FFA delivery from VAT lipolysis correlates positively with visceral fat mass in lean and obese subjects. (8, 9, 10) While whole-body systemic FFA availability is similar in obese individuals with and without NAFLD, those with NAFLD have greater hepatic VLDL-TG secretion. (11, 12, 13) It is unclear if the proportion of hepatic FFA delivery from VAT lipolysis is greater in obese adults with NAFLD than those without. If so, VAT lipolysis could play a role in IHTG accumulation and the progression to NASH. Additionally, altered hepatic VLDL-TG secretion and increased splanchnic TG uptake may also contribute to the development of simple steatosis and NASH.

In the present study, we wanted to establish whether randomly selected groups with simple steatosis and NASH were different with respect to splanchnic FFA and VLDL-TG balances, because if this is the case, then treatment approaches should most likely also be different. We hypothesized that splanchnic FFA uptake is greater and VLDL-TG export lower in obese men with NASH than obese men with simple steatosis when matched for body fat. We further hypothesized that these differences exist despite similar systemic turnover of FFA in obese men with simple steatosis and NASH. We examined these hypotheses during fasting and hyperinsulinemia.

## Materials and methods

Written informed consent from all participants and Regional Ethics Committee approval (N^o^ 1-10-72-283-18) were obtained before the study, which was registered at www.clinicaltrials.gov (NCT04292977) and conducted in accordance with both the Declaration of Helsinki.

### Study-design

Obese men (BMI >28 kg/m^2^) with NAFLD (liver fat fraction (FF%) >5.6%) and no history of liver disease, diabetes, or dyslipidemia were recruited through newspaper advertisements. Screening comprised clinical examination, electrocardiogram, and blood samples to document normal blood and chemistry panels, except moderately elevated alanine aminotransferase. Additional criteria were aged 40–70 years, nonsmokers, alcohol intake <21 units/week, weight stable (self-reported ± 5%) for >3 months, and not engaged in strenuous exercise. Eligible subjects had an NMR spectroscopy to ascertain the presence of NAFLD and an MRI to measure abdominal fat distribution. A fibroscan was performed to measure liver stiffness. Subjects with documented NAFLD were invited, and accepted, to have a liver biopsy performed to determine the presence of simple steatosis or NASH, including NASH severity. To minimize unnecessary biopsies towards the end of the recruitment period, which was during the Covid-19 pandemic, the fibroscan was employed to assess the probability of NASH or simple steatosis. Using this approach, only one additional biopsy was performed (1 NASH) and it was decided to include this subject in the study. We interviewed each volunteer about their body weight history and reviewed their medical records for logged body weight and liver function test from 2000 to 2019–2020.

### Patients

Nine men with NASH and eight men with simple steatosis were included. Three were treated for hypertension, two with statins (held for 14 days before the study), and two took fish oil (held for 1 month before the study). All consumed a weight-maintaining diet (55% carbohydrate, 15% protein, 30% fat) provided by the hospital kitchen for three days prior to the study. The diet was designed by a dietitian, who interviewed each volunteer to assure the diet was individualized.

### Liver morphology

Ultrasonographic-guided liver biopsies were performed within a few weeks of the study day by an experienced hepatologist and recorded by a proficient pathologist and in agreement with recognized principles. (14, 15) Presence of >5% steatosis in combination with both hepatocyte ballooning and lobular inflammation was used to define NASH, whereas steatosis combined with the absence of ballooning and/or inflammation defined simple steatosis. (14) To determine the liver FF%, we used NMR (Siemens Skyra, 3T MR scanner) in a single 3x2x2 cm^3^ voxel as reported earlier. LCModel software package version 6.3-1L (Stephen Provencher, 2016) was used to analyze data.

### Protocol

One week before the study blood samples were taken after a 12-h fast under sterile conditions for VLDL-TG, tracer preparation and a dual energy-x-ray absorptiometry scan was performed. Participants were admitted to the Clinical Research Center the evening before the study. From then, only mineral water was permitted, and they remained in bed under thermoneutral conditions. Next morning, catheters for infusion were placed in an antecubital vein and in a contralateral heated hand vein to obtain arterialized blood. A catheter was placed in the right femoral artery for arterial blood sampling and an additional catheter was placed through the right femoral vein into the hepatic vein for collection of blood samples. The hepatic catheter was kept patent with small amounts of heparin, which was aspirated before sampling, and placement was monitored by regular x-ray imaging using contrast infusion.

**Fig. 1:**
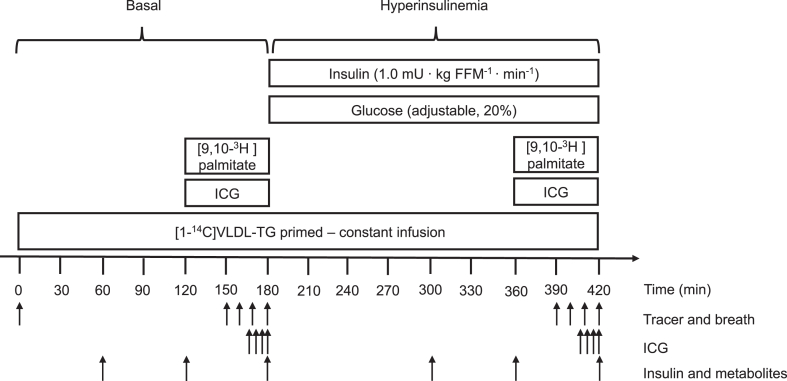
Metabolic study day. Basal (0–180) minutes and hyperinsulinemic clamp (180–420) minutes periods. ICG, indocyanine green.

The study day comprised a 3-h basal period and a 4-h hyperinsulinemic, euglycaemic clamp (Fig. 1). From 180-420 min, human insulin (Humulin; Eli Lilly) was infused (1.0 mU⋅kg FFM^-1^⋅min^-1^). (16) Blood glucose was measured every 10 min during the insulin infusion and clamped at ∼5 mmol/L using a variable infusion of 20% glucose. The average glucose infusion rate during the last 30 min of the clamp was used as a measure of insulin sensitivity. Indirect calorimetry (Deltatrac monitor, Datex Instrumentarium, Helsinki, Finland) was used to measure resting energy expenditure and respiratory exchange ratio. A 1-h constant infusion of [9,10-^3^H]palmitate was given during the last hour of each period to measure palmitate turnover. A primed-constant infusion of [1–^14^C]triolein-labeled VLDL-TG (20% bolus, 80% constant) was administered during the whole study period to measure VLDL-TG kinetics. Indocyanine green (ICG) was infused (90 ml h^−1^) intravenously for one hour to measure hepatic venous plasma flow during both periods. Blood samples for plasma palmitate concentration and specific activity (SA) and for plasma VLDL-TG concentration and SA were collected with ^14^CO_2_ in breath samples every 10 min during the last 30 min of each period. Blood samples for ICG concentrations were collected from 165-180 and 405-420 min. Insulin and metabolite concentrations were measured regularly. One subject (NASH) did not complete the clamp due to nausea. Splanchnic plasma flow was unavailable in one subject with NASH (clamp) and one subject with simple steatosis (basal and clamp) due to technical issues. Thus, 16 subjects (8 NASH, 8 simple steatosis) completed the full systemic palmitate and VLDL-TG turnover part of the study. Fourteen subjects (7 NASH, 7 simple steatosis) completed the full study protocol. No statistical difference was observed in kinetic parameters at baseline between the full study group and those who completed the whole study. After completion of the study, all catheters were removed, the volunteers were observed for 2 h, had lunch, and were discharged after stable plasma glucose was ensured.

### Palmitate concentration and SA

A 1-h constant infusion of [9,10-^3^H]palmitate (0.3 μCi/min; Department of Clinical Physiology and Nuclear Medicine, Aarhus University Hospital, Denmark) was employed to measure systemic palmitate turnover and splanchnic balances. Plasma palmitate concentration and SA were measured in arterial and hepatic venous blood by HPLC. Steady state SA was verified for everyone.

### VLDL-TG tracer preparation

An 80-ml blood sample was taken under sterile conditions for VLDL-TG labeling as described previously, (17) with minor modifications. Plasma was immediately separated and sonicated with 20 μCi [1–^14^C]triolein (PerkinElmer, Waltham, MA) at 5°C for 2 h in sterile test tubes. The [1-^14^C]triolein-labeled plasma was transferred to sterile tubes, covered with a sterile saline (d = 1.006 g/cm^3^), and ultracentrifuged (50.3 Ti rotor (40.000 rpm) or 50.4 Ti rotor (37.000 rpm), Beckman Instruments (Palo, Alto, CA)) for 18 h at 4°C. The supernatant was collected with a sterile Pasteur pipette, passed through a 0.20 μm Millipore filter, and stored under sterile conditions at 5°C. A small sample was cultured to ensure sterility before autologous infusion.

### Plasma VLDL-TG concentration and SA

VLDL-TG was separated from ∼3 ml weighed arterial and hepatic vein samples by ultracentrifugation as described above. VLDL was obtained by tube slicing (Beckman Instruments) ∼1 cm from the top and the exact volume recorded. VLDL-TG content was analyzed in 300 μl aliquots and the plasma concentration calculated. The remaining VLDL-TG was transferred to scintillation glasses and [^3^H] and [^14^C] activity measured by dual channel scintillation counting to <2% counting error. Steady state SA was verified for everyone.

### Breath ^14^CO_2_ SA

Breath samples were collected in breath bags (IRIS-breath-bags; Wagner Analysen Technik) to calculate [1–^14^C]VLDL-TG fatty acid oxidation by using the activity of ^14^CO_2_ in expired air as described in detail previously (11).

### Body composition

Subcutaneous abdominal fat and VAT mass were quantified using MRI. Fat mass was calculated as the volume multiplied by the average density of TG (0.900 kg/L). We used dual energy-x-ray absorptiometry scanning (QDR-2000, Hologic Marlborough, Ma) to measure total fat mass, fat percentage, and fat-free mass.

### Laboratory procedures

Blood samples were quickly separated by centrifugation. Triglyceride concentrations were measured on a Cobas 111 using glycerol blanked kits (F. Hoffmann-La Roche). Plasma ICG was measured using spectrophotometry. Serum insulin concentrations were determined with an immunoassay (DAKO Denmark A/S), serum NEFA concentrations by a colorimetric method (Wako Pure Chemical Industries), and plasma glucose on a YSI 2.300 STAT Plus analyzer.

### Calculations

Palmitate turnover (μmol/min) was calculated as [9,10-^3^H]palmitate infusion rate divided by the steady-state palmitate SA. (18, 19, 20) Steady-state palmitate concentrations and SA were combined with splanchnic plasma flow to calculate splanchnic palmitate uptake and release rates as described previously. (21) The percent of palmitate delivered to the liver that originated from VAT lipolysis was calculated as reported previously. (22) Hepatic VLDL-TG secretion was calculated as [1–^14^C]VLDL-TG infusion rate divided by the steady-state VLDL-TG SA. Because all circulating VLDL-TG originates from the liver, splanchnic VLDL-TG release equal systemic VLDL-TG turnover. Splanchnic VLDL-TG uptake was calculated by combining splanchnic VLDL-TG delivery and exit rates (VLDL-TG concentration times plasma flow) with the isotopically determined VLDL-TG secretion rate: (VLDL uptake = VLDL-TG delivery - VLDL-TG exit + VLDL-TG secretion). To calculate VLDL-TG oxidation, the VLDL-TG secretion rate was multiplied by the fractional oxidation of the infused VLDL-TG tracer as (^14^CO_2_SA⋅VCO_2_)/(k⋅Ar⋅F), where k is the volume of CO_2_ at 20°C and 1 atm. pressure (22.4 l/mol), Ar is the fractional acetate carbon recovery factor in breath CO_2_ (0.56 at rest and 0.5 for hyperinsulinemia), (23) and F is the tracer infusion rate.

### Statistics

Sample size was based on VLDL-TG secretion, for which we have extensive data on population means and variability. We calculated a sample of 8 in each group to detect a 20% change (α = 0.05, β = 0.80) in VLDL-TG secretion between groups at baseline and within-group change from baseline to hyperinsulinemia. Data were analyzed with SigmaPlot 14.0 and SPSS 27 and presented as mean ± SD or 95% CI or as the median (range). Concentrations and kinetic data are presented as the average value of each period. To assess the effect of insulin on total plasma FFA, glucose, and insulin concentrations, we used a two-way analysis of variance for repeated measurements (RM-ANOVA), and for the kinetic data, a linear Mixed Model analysis with factor variables for group and time and group versus time interaction. Between and within group comparisons and correlations were tested using parametric or nonparametric test as appropriate. A *P*-value <0.05 was considered significant for the two main splanchnic kinetic parameters (splanchnic palmitate uptake and splanchnic VLDL-TG release), whereas a *P*-value <0.01 was considered significant for all other splanchnic kinetic parameters. For the remaining (nonsplanchnic) parameters, a *P*-value <0.05 was considered significant.

## Results

Subject characteristics are shown in Table 1. We observed an age difference between groups which was not unexpected; men with NASH were older. (24) All other baseline characteristics were not significantly different. The liver histology for each participant is shown in Table 2. The Steatosis-Activity-Fibrosis score and NAFLD Activity Score performed similarly regarding diagnosis of simple steatosis versus NASH. We found no difference in obesity duration or liver function. In addition, there was no significant relationship between age and our splanchnic kinetic endpoints.

**Table 1:** Subject characteristics

	NASH	Simple Steatosis	*p*
N	9	8	
Age (years)	64 (6)	55 (5)	<0.01
BMI (kg/m^2^)	34.1 (3.9)	32.2 (2.2)	0.22
FF%	14.5 (8.2–31.6)	9.4 (6.2–51.0)	0.27
Body fat (%)	40 (5)	39 (3)	0.53
VAT (kg)	8.4 (4)	9.0 (2)	0.69
SAT (kg)	10.4 (3)	9.6 (2)	0.47
FFM (kg)	63.6 (7.7)	62.9 (6.0)	0.83
HbA_1c_ (%)	36 (3)	34 (4)	0.27
Triglycerides (mmol/L)	1.5 (0.5)	2.1 (1.0)	0.17
Total cholesterol (mmol/L)	4.3 (4.1–7.0)	5.1 (4.3–7.2)	0.16
LDL-cholesterol (mmol/L)	3.0 (1.0)	3.2 (0.5)	0.76
HDL-cholesterol (mmol/L)	1.12 (0.21)	1.17 (0.29)	0.70
ALT (U/L)	52 (36)	35 (8)	0.20
REE (kcal/day)	1991 (232)	1948 (250)	0.72
RER	0.81 (0.02)	0.80 (0.04)	0.43

Values are mean (SD) or median (range). Students *t* test or Mann-Whitney two-sample test.

ALT: alanine aminotransferase; FF%: fat fraction; FFM: fat free mass; REE: resting energy expenditure; RER: respiratory exchange ratio; SAT: subcutaneous adipose tissue; VAT: visceral adipose tissue.

**Table 2:** Liver histology

Subject Id	SAF Score	NAFLD Activity Score	Diagnosis
1	S1 A2 (1+1) F1a	3 (1+1+1) F1a	NASH
2	S2 A2 (1+1) F2	4 (2+1+1) F2	NASH
3	S2 A2 (1+1) F1a	4 (2+1+1) F1a	NASH
4	S3 A2 (1+1) F1a	5 (3+1+1) F1a	NASH
5	S2 A2 (1+1) F1a	4 (2+1+1) F1a	NASH
6	S2 A2 (1+1) F2	4 (2+1+1) F2	NASH
7	S2 A2 (1+1) F1a	4 (2+1+1) F1a	NASH
8	S2 A4 (2+2) F1a	6 (2+2+2) F1a	NASH
9	S2 A2 (1+1) F1a	4 (2+1+1) F1	NASH
10	S2 A1 (1+0) F0	3 (2+1+0) F0	Simple steatosis
11	S3 A1 (0+1) F0	4 (3+0+1) F0	Simple steatosis
12	S2 A1 (0+1) F0	3 (2+0+1) F0	Simple steatosis
13	S1 A0 (0+0) F0	1 (1+0+0) F0	Simple steatosis
14	S1 A0 (0+0) F0	1 (1+0+0) F0	Simple steatosis
15	S1 A1 (1+0) F1a	2 (1+1+0) F1a	Simple steatosis
16	S2 A1 (0+1) F1a	3 (2+0+1) F1a	Simple steatosis
17	S1 A0 (0+0) F0	1 (1+0+0) F0	Simple steatosis

All biopsies were reviewed by a proficient liver pathologist, who was blinded with respect to the subjects. The SAF score by Bedossa (14) and The NAFLD Activity score (NAFLD Activity Score) by Kleiner (15) were calculated in all subjects.

Steatosis-Activity-Fibrosis score: S: steatosis (grade S0-S3); A: activity (grade 0–4 (ballooning (grade A0-A2) + inflammation (grade A0-A2)); F: fibrosis (grade F0, F1(a,b,c), F2, F3, F4).

NAFLD Activity Score: Numbers in parenthesis are as follows: steatosis (grade 0–3) + ballooning (grade 0–3) + inflammation (grade 0–2). F: fibrosis (grade F0, F1, F1(a,b,c,), F2, F3, F4).

### Hormones and metabolites

**Fig. 2:**
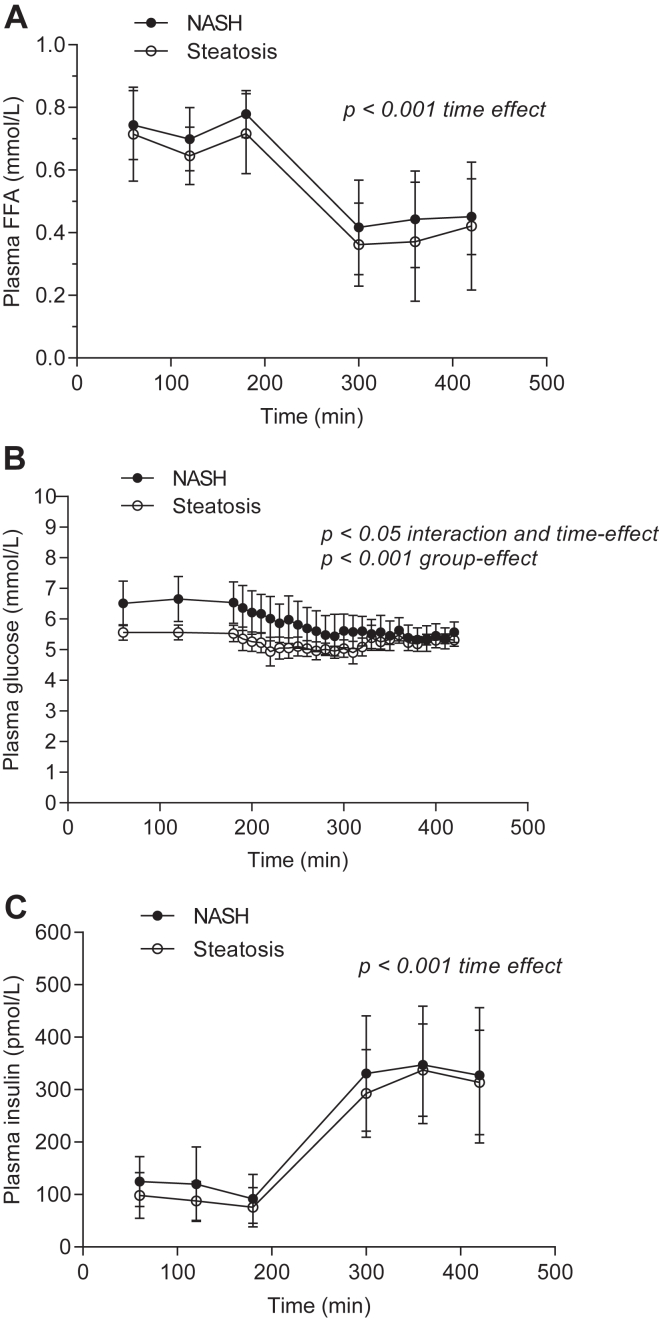
Hormone and metabolite concentrations. (A) FFA, (B) glucose, (C) insulin in the basal (60–180 min) and hyperinsulinemic clamp (180–420 min) periods. Data are mean (95% CI). (A): *P* = 1.00 interaction (group × time (60–420 min), *P* = 0.12 group-effect, ∗*P* < 0.001 time-effect (60–420 min), Mixed Model; (B) ∗*P* = 0.03 interaction (group x time), ∗∗*P* < 0.001 group-effect, ∗*P* = 0.02 time-effect (60–420 min), Mixed Model; (C), *P* = 1.00 interaction (group × time (60–420 min)), *P* = 0.22 group-effect, ∗*P* < 0.001 time-effect (60–420 min), Mixed Model. *Black circles*, NASH; *white circles*, simple steatosis.

Concentrations of FFA, glucose, and insulin are presented in Fig. 2A–C. Basal FFA concentrations were similar in the two groups and were similarly suppressed during hyperinsulinemia. Insulin concentrations were not different between groups. To test whether the missing values created bias, we compared the basal values of the entire study group (9 NASH; 8 simple steatosis) with the basal values of the subjects who completed the study; there was no statistical difference.

### Systemic palmitate kinetics

Systemic palmitate turnover, a measure of adipose tissue lipolysis, was comparable between groups and was equally suppressed during hyperinsulinemia (NASH: 345 (257–434) versus 253 (184–323) μmol/min; simple steatosis: 327 (234–421) versus 232 (162–301) μmol/min mean (95% CI), basal versus clamp periods, respectively, *P* = 0.97 interaction (group x time (basal vs. clamp)), *P* = 0.60 group-effect, *P* = 0.02 time-effect (basal vs. clamp), Mixed Model).

### Splanchnic palmitate kinetics

Basal and insulin stimulated splanchnic plasma flow was not significantly different between the two groups (NASH: 722 (646–798) versus 817 (652–982) ml/min; simple steatosis: 690 (613–766) versus 803 (638–968) ml/min, mean (95% CI), basal versus clamp periods, respectively, *P* = 0.87 interaction (group x time (basal vs. clamp)), *P* = 0.70 group-effect, *P* = 0.09 time-effect (basal vs. clamp)), Mixed Model).

**Fig. 3:**
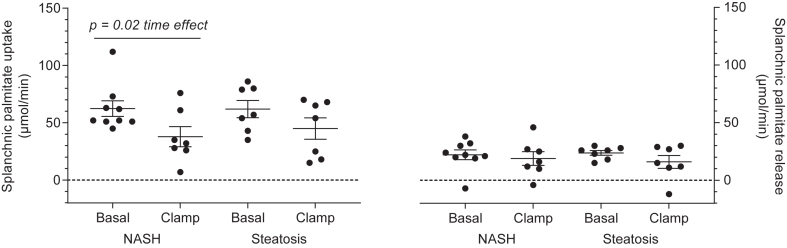
Splanchnic palmitate kinetics. Splanchnic palmitate uptake rate (*left* panel) and splanchnic palmitate release rate (*right* panel) during the basal hyperinsulinemic clamp periods. Data are mean ± SEM. Splanchnic uptake rate, *P* = 0.65 interaction (group × time (basal versus clamp), *P* = 0.68 group-effect, ∗*P* = 0.02 time-effect (basal vs. clamp), Mixed Model; Splanchnic release rate, *P* = 0.65 interaction (group × time (basal vs. clamp)), *P* = 0.90 group-effect, *P* = 0.27 time-effect (basal vs. clamp), Mixed Model. *Black* bars, NASH; *white* bars simple steatosis.

The splanchnic uptake of arterially delivered palmitate was similar in the two groups (Fig. 3). Hyperinsulinemia resulted in a similar significant decrease in splanchnic palmitate uptake in the two groups (NASH: 62 (48–77) versus 38 (18–58) μmol/min; simple steatosis: 62 (46–78) versus 45 (25–65) μmol/min, mean (95% CI), basal versus clamp periods, respectively, *P* = 0.65 interaction (group x time (basal vs. clamp)), *P* = 0.68 group-effect, *P* = 0.02 time-effect (basal vs. clamp), Mixed Model).

We examined whether the change in splanchnic palmitate uptake between the basal and hyperinsulinemic periods was a function of different palmitate delivery to the splanchnic bed or was due to different fractional extraction of palmitate across this tissue bed. Splanchnic palmitate delivery decreased significantly during the insulin clamp but was not different between groups (NASH: 137 (112–163) versus 86 (53–119) μmol/min; simple steatosis: 131 (101–160) versus 105 (72–138) μmol/min, mean (95% CI), basal versus clamp periods, respectively, *P* = 0.37 interaction (group x time (basal vs. clamp)), *P* = 0.66 group-effect, *P* = 0.01 time-effect (basal vs. clamp), mixed Model). The percent of systemically delivered palmitate taken up by the splanchnic bed was not significantly different between groups (NASH: 47 (39–54) versus 41 (28–54)%; simple steatosis: 48 (40–57) versus 45 (32–58)%, mean (95% CI), basal versus clamp periods, respectively, *P* = 0.79 interaction, *P* = 0.54 group-effect, *P* =0.37 time-effect (basal vs. clamp), Mixed Model).

Splanchnic palmitate release was not significantly different between groups and was similarly, but not significantly, reduced during hyperinsulinemia (NASH: 22 (15–29) versus 19 (6–31) μmol/min; simple steatosis: 24 (15–32) versus 16 (3–29) μmol/min, mean (95% CI), basal versus clamp periods, respectively, *P* = 0.65 interaction (group vs. time (basal vs. clamp)), *P* = 0.90 group-effect, *P* = 0.27 time-effect (basal vs. clamp), Mixed Model) (Fig. 3). The percent of systemic palmitate release originating from the splanchnic bed was not significantly different between the groups (NASH: 7 (4–10) versus 8 (3–13)%; simple steatosis: 8 (5–11) versus 8 (3–13)%, mean (95% CI), basal versus clamp periods, respectively, *P* = 0.98 interaction, *P* = 0.74 group-effect, *P* = 0.86 time-effect (basal vs. clamp), Mixed Model). We also calculated the percent of palmitate delivered to the liver that originated from VAT lipolysis and found it to be similar in the two groups and not altered significantly during hyperinsulinemia (NASH: 23 (13–32) versus 27 (12–42)%; simple steatosis: 27 (17–37) versus 29 (14–44)%, mean (95% CI), basal versus clamp periods, respectively, *P* = 0.83 interaction (group x time (basal vs. clamp)), *P* = 0.62 group-effect, *P* = 0.62 time-effect (basal vs. clamp), Mixed Model).

### Systemic and splanchnic VLDL-TG kinetics

The arterial plasma VLDL-TG concentrations were comparable in the two groups and equally and significantly suppressed during hyperinsulinemia (NASH: 0.98 (0.60–1.36) versus 0.65 (0.31–0.99) mmol/L; simple steatosis: 0.95 (0.54–1.35) versus 0.82 (0.48–1.17) mmol/L, mean (95% CI), basal versus clamp, respectively, *P* = 0.55 interaction (group x time (basal vs. clamp)), *P* = 0.70 group-effect, *P* = 0.004 time-effect (basal vs. clamp), Mixed Model).

**Fig. 4:**
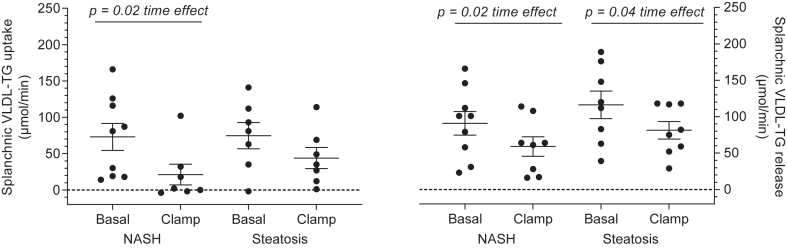
Splanchnic VLDL-TG kinetics. Splanchnic VLDL-TG uptake rate (*left* panel) and splanchnic VLDL-TG release rate (*right* panel) during the basal and hyperinsulinemic clamp periods. Data are mean ± SEM. Splanchnic uptake rate, *P* = 0.54 interaction (group x time (basal vs. clamp)), *P* = 0.47 group-effect, ∗*P* = 0.02 time-effect (basal vs. clamp), Mixed Model; Splanchnic release rate, *P* = 0.92 interaction (group × time (basal vs. clamp)), *P* = 0.13 group-effect, ∗∗*P* = 0.04 time-effect (basal vs. clamp), Mixed model. *Black* bars, NASH; *white* bars simple steatosis.

The splanchnic uptake of VLDL-TG was similar in the two groups (Fig. 4) and was significantly reduced during the clamp (NASH: 73 (35–111) versus 21 (-10-53) μmol/min; simple steatosis: 75 (32–117) versus 44 (12–75) μmol/min, mean (95% CI), basal versus clamp periods, respectively, *P* = 0.54 interaction (group x time (basal vs. clamp)), *P* = 0.47 group-effect, *P* = 0.02 time-effect (basal vs. clamp)), Mixed Model).

The splanchnic VLDL-TG release was also similar in the two groups (Fig. 4) and similarly suppressed during hyperinsulinemia (NASH: 91 (55–127) versus 59 (32–86) μmol/min; simple steatosis: 116 (78–154) versus 81 (54–109) μmol/min, mean (95% CI), basal versus clamp, respectively, *P* = 0.92 interaction (group x time (basal vs. clamp)), *P* = 0.13 group-effect, *P* = 0.04 time-effect (basal vs. clamp), Mixed model).

The VLDL-TG oxidation rate was not significantly different between groups and was not significantly altered during hyperinsulinemia (NASH: 34 (20–48) versus 34 (18–51) μmol/min; simple steatosis: 38 (23–53) versus 43 (26–59) μmol/min, mean (95% CI), basal versus clamp periods, respectively, *P* = 0.76 interaction (group x time (basal vs. clamp)), *P* = 0.40 group-effect, *P* = 0.74 time-effect (basal vs. clamp), Mixed-Model).

#### Correlations

FF% did not correlate significantly with basal or clamp palmitate and VLDL-TG uptake rates or VLDL-TG release rate in any of the groups. In addition, the basal and clamp percent palmitate delivery to the liver from visceral fat lipolysis did not correlate significantly with splanchnic VLDL-TG release rate in any of the groups.

#### Systemic glucose kinetics

Glucose infusion rate was similar in subjects with NASH compared with simple steatosis: (1.6 (0.5–2.7) versus 2.2 (0.4–4.0) mg⋅kg FFM^−1^⋅min^−1^, respectively, mean (95% CI), *P* = 0.54, Student’s *t* test).

## Discussion

This study aimed to investigate whether there are gross abnormalities in splanchnic lipid balance between obese men with biopsy-proven NASH and simple steatosis because therapeutic approaches could be different depending on whether such differences do exist. The study used a validated model combining blood sampling with FFA and VLDL-TG tracers to compare splanchnic lipid balance during basal and hyperinsulinemic conditions. The results revealed no significant difference in splanchnic lipid balance between subjects with NASH and simple steatosis, with similar splanchnic palmitate and VLDL-TG uptake and release rates.

The study showed that splanchnic uptake of systemically delivered palmitate was not significantly different in men with NASH compared with simple steatosis and that the palmitate uptake was similarly and significantly reduced during hyperinsulinemia. In addition, the results revealed that both splanchnic palmitate delivery and the fractional uptake of palmitate from plasma in the splanchnic bed were similar in the two groups. We are not aware of other studies that have investigated these questions in subjects with NASH versus simple steatosis being either biopsy-proven or assessed by noninvasive measures. We also tested whether the percent of hepatic palmitate delivery that originated from VAT lipolysis is greater in NASH than simple steatosis, because this represents an additional source of hepatic FFA exposure that cannot be measured by other methods. We found no significant difference between the two groups. Thus, visceral fat mass, which was of similar size in the two groups, contributed an equivalent fraction of total hepatic palmitate delivery. This implies that increased VAT lipolysis relative to fat mass is not likely to explain the differences between NASH and simple steatosis pathophysiology. In this context, however, we previously reported that the relative contribution of VAT lipolysis to hepatic FFA delivery increases with increasing visceral fat mass ranging from 14% to 25% in lean and upper body obese individuals, respectively and a further increase to ∼55% during a high-dose hyperinsulinemic clamp (9, 10), which supports the notion that visceral fat lipolysis impacts directly on the adverse hepatic metabolic manifestations of obesity.

Splanchnic palmitate release was also similar in subjects with NASH and simple steatosis and contributed equally to systemic palmitate turnover during fasting and hyperinsulinemia. In addition, there was no difference in systemic palmitate turnover between the two groups during fasting or hyperinsulinemia. In contrasts to previous findings, the present study did not show that the relative contribution of splanchnic palmitate release to systemic palmitate turnover increases during hyperinsulinemia (25, 26) but remain unchanged (∼8%). Although a lean control group was not included, we speculate that the presence of NAFLD is associated with more pronounced insulin resistance with respect to anti-lipolysis of non-VAT compared with what was found earlier. Palmitate turnover was indeed somewhat greater in the present study compared with what we typically find in obese men using the same insulin infusion protocol and less challenging study designs. (11, 16, 27).

Basal splanchnic uptake of VLDL-TG was similar in the two groups and was not differently affected by hyperinsulinemia. Of importance, while splanchnic release of VLDL-TG only occurs in the liver, splanchnic VLDL-TG uptake takes place in both liver and nonhepatic splanchnic tissues, especially VAT. We previously reported that the VLDL-TG uptake per gram fat in the VAT of viscerally obese subjects was similar to the uptake rates that we have found in upper-body nonsplanchnic fat. (28, 29) In addition, for the whole VAT depot, the fractional uptake was very low, indicating that the majority of VLDL-TG that traverses the splanchnic bed ultimately reaches the liver. Therefore, we do not believe that differences in adipose tissue VLDL-TG uptake can account for major differences in hepatic VLDL-TG delivery.

No difference in splanchnic VLDL-TG release rate between NASH and simple steatosis was found in the study. Both groups showed a similar significant suppression of VLDL-TG release under hyperinsulinemia. We previously reported that obese men with increased IHTG content are more resistant to insulin suppression of VLDL-TG secretion. (11) The finding that hepatic lipid export in VLDL particles is not different between men with NASH and simple steatosis therefore suggests that other pathways than lipid export must be involved in the progression towards NASH. However, as NASH activity may not be uniformly distributed within the liver, we cannot exclude that tissue areas with normal architecture can compensate and sustain normal VLDL-TG secretion. Nonetheless, the overall splanchnic lipid uptake and release rates were remarkably similar between groups, implying that abnormalities in splanchnic/hepatic lipid metabolism may not be determinants of progression to NASH and that intervention against other factors, such as inflammation, may be more beneficial.

Subjects with NASH and simple steatosis were similarly insulin-resistant with respect to glucose disposal. We did not use a glucose tracer in the present study, and we therefore cannot determine whether our groups differed in insulin sensitivity to endogenous glucose production. We are unaware of published studies that have compared insulin sensitivity to glucose turnover between patients with biopsy-proven NASH and simple steatosis using the gold standard hyperinsulinemic euglycaemic clamp approach, and we therefore consider our results as novel information. In a study using a glucose tracer in subjects with normal and increased IHTG, it was reported that elevated IHTG was associated with a reduction in both insulin-mediated glucose disposal and endogenous glucose production. (30) However, the reduced insulin sensitivity was not related to the degree IHTG content among the subjects with increased IHTG. However, liver biopsy data were not provided for those that underwent the clamp studies.

Both the duration (24) and the severity (31) of obesity are independent, strong predictors of progression to NASH. We found no signs of different obesity duration or liver function tests between groups over a 20 year period before the study. In addition, no relationship between age and our primary end points was noted. A central element of our design was to recruit community living volunteers with no prior knowledge about their liver fat status and who were otherwise healthy (except hypertension and hypercholesterolemia). This led to a significant age difference between groups that was not unexpected, as studies have shown that subjects with NASH are generally older than those with simple steatosis. (24) Our key research question was whether gross abnormalities of splanchnic lipid uptake and release rates existed between subjects with simple steatosis and NASH. If such differences were observed, it would suggest they should be therapeutically targeted. However, longer obesity duration could cause obesity-related effects on hepatic lipid metabolism and TG accumulation leading to progression to NASH, and treatment interventions to lower hepatic lipid delivery could potentially lead to less disease progression with impact on hepatic lipid metabolism.

There are limitations to our study. First, the limited number of participants due to the elaborate and costly design could result in type 2 errors. However, we have previously demonstrated significant differences in palmitate and VLDL-TG turnover with a similar number of participants. Second, we investigated only men due to the well-known differences in lipid metabolism between sexes, (9, 20, 32), so our results cannot be extended to women. Third, the assessment of hyperinsulinemia using a single step infusion design may not reveal differences in splanchnic palmitate and VLDL-TG kinetics at different insulin infusion rates. However, we previously showed that a single insulin clamp infusion of 1.0 mU/kg fat-free mass per minute allows a simultaneous comparison of insulin regulation of lipolysis and glucose metabolism and that the effect on FFA correlates with the effect on TG concentrations. (16) Fourth, our tracer method does not allow differentiation between the splanchnic chylomicron-remnant-TG and VLDL-TG balances as both lipoproteins appear in the same ultracentrifugation fraction (Svedberg flotation rate 20–400) during both the labeling and study day procedures. Hence, whether postprandial hepatic and/or adipose tissue uptake of meal derived chylomicron-remnant-TG and VLDL-TG are similar and whether they are comparable between groups cannot be inferred. The strengths of our study include well-phenotyped individuals, liver biopsy–documented conditions, and state-of-the-art measurements of hepatic perfusion and validated tracer kinetics. In this study, we found that splanchnic palmitate and VLDL-TG uptake and release rates are similar in men with NASH and simple steatosis during fasting and hyperinsulinemia. The study also showed that the fractional extraction of palmitate in the splanchnic bed and the percent of total hepatic palmitate delivery from visceral fat lipolysis are comparable between the two groups during fasting and hyperinsulinemia. However, splanchnic palmitate uptake and VLDL-TG release are significantly suppressed during hyperinsulinemia, while the insulin-mediated suppression of splanchnic palmitate release and VLDL-TG uptake are less modified. Therefore, it can be concluded that the development of NASH is likely independent of splanchnic/hepatic FFA metabolism and that treatment aimed at addressing other components, such as inflammation, may be more beneficial.

## Data availability

Data are available from the corresponding author upon reasonable request.

## Conflicts of interest

The authors declare that they have no conflicts of interest with the contents of this article.
